# Epigenetic Mechanisms and Therapeutic Targets in Chemoresistant High-Grade Serous Ovarian Cancer

**DOI:** 10.3390/cancers13235993

**Published:** 2021-11-29

**Authors:** Bayley G. Matthews, Nikola A. Bowden, Michelle W. Wong-Brown

**Affiliations:** 1 Centre for Drug Repurposing and Medicines Research, University of Newcastle, Newcastle, NSW 2289, Australia; Bayley.Matthews@uon.edu.au (B.G.M.); Nikola.Bowden@newcastle.edu.au (N.A.B.); 2 School of Medicine and Public Health, University of Newcastle, Newcastle, NSW 2289, Australia; 3 Hunter Medical Research Institute, Newcastle, NSW 2289, Australia; 4 School of Biomedical Sciences and Pharmacy, University of Newcastle, Callaghan, NSW 2308, Australia

**Keywords:** high-grade serous ovarian cancer, chemoresistance, epigenetic modifications, DNA methylation, histone acetylation, microRNA, DNA methyltransferase inhibitors, histone deacetylase inhibitors

## Abstract

**Simple Summary:**

High-grade serous ovarian cancer (HGSOC) is the most common ovarian cancer subtype. While 60–80% of HGSOC patients initially respond to treatment, the majority of patients will eventually become platinum resistant. Epigenetic modifications are mechanisms that alter the expression of a gene but do not change the DNA sequence itself. Several types of epigenetic modifications, including DNA methylation, histone deacetylation, and microRNA expression, have been implicated in the progression of HGSOC to chemoresistance. These modifications can be targeted by epigenetic modulating therapies to overcome chemoresistance. This review summarises the epigenetic modifications identified in chemoresistant HGSOC and clinical trials utilizing epigenetic therapies in HGSOC.

**Abstract:**

High-grade serous ovarian cancer (HGSOC) is the most common ovarian cancer subtype, and the overall survival rate has not improved in the last three decades. Currently, most patients develop recurrent disease within 3 years and succumb to the disease within 5 years. This is an important area of research, as the major obstacle to the treatment of HGSOC is the development of resistance to platinum chemotherapy. The cause of chemoresistance is still largely unknown and may be due to epigenetics modifications that are driving HGSOC metastasis and treatment resistance. The identification of epigenetic changes in chemoresistant HGSOC enables the development of epigenetic modulating drugs that may be used to improve outcomes. Several epigenetic modulating drugs have displayed promise as drug targets for HGSOC, such as demethylating agents azacitidine and decitabine. Others, such as histone deacetylase inhibitors and miRNA-targeting therapies, demonstrated promising preclinical results but resulted in off-target side effects in clinical trials. This article reviews the epigenetic modifications identified in chemoresistant HGSOC and clinical trials utilizing epigenetic therapies in HGSOC.

## 1. Chemoresistance in HGSOC

Standard-of-care treatment for high-grade serous ovarian cancer (HGSOC) consists of combination carboplatin and paclitaxel chemotherapy. While 60–80% of HGSOC patients initially respond to treatment, the majority of patients will eventually become platinum resistant [1]. Although the exact mechanisms of platinum resistance are still unknown, cancer stem cells, epithelial-to-mesenchymal transition, and dysfunctional DNA repair pathways are thought to aid in the development of chemoresistance in HGSOC [2,3,4].

Cancer stem cells (CSCs) are a small subgroup of cancer cells which are characterised by their ability to self-renew and give rise to both CSCs and non-CSCs within a heterogenous tumour [2]. CSCs have been identified as the most treatment-resistant cells within tumours and have been linked to the development of platinum resistance in HGSOC [2]. One likely mechanism of this chemoresistance is the ability of CSCs to be quiescent for long periods [5]. As chemotherapy relies on cell division to damage DNA, quiescent CSCs remain unaffected by therapy and allow for disease recurrence.

Epithelial-to-mesenchymal transition (EMT) is a process by which epithelial cells lose their epithelial characteristics and take on properties of mesenchymal cells, including disruption of adhesions to other cells and the cellular basement membrane, as well as increased cell migration and invasiveness [6,7]. Studies have previously shown that the activation of EMT also confers properties seen in CSCs, indicating that EMT activation is closely linked to the development of CSCs [8,9]. EMT also shares many signalling pathways to CSCs, including Wnt and Hedgehog pathways [10,11]. While HGSOC develops from epithelial cells, platinum-resistant tumours often display characteristics of mesenchymal cells [3], indicating a potential role of EMT in acquisition of chemoresistance. However, the exact mechanism of EMT in chemoresistant HGSOC is still unknown.

Defective DNA repair pathways such as the homologous recombination (HR) and nucleotide excision repair (NER) pathways are also crucial in the development of chemoresistance in HGSOC. The HR pathway is involved in repairing double-strand breaks that occur at sites of DNA crosslinks caused by platinum chemotherapy during DNA replication [4,6]. This pathway is regulated by BRCA1 and BRCA2 proteins that are involved in homologous recombination, which are deficient in approximately 50% of HGSOC patients [12], and results in increased double-strand breaks (DSBs) after platinum chemotherapy. While this initially increases sensitivity to platinum treatments [13], HR-deficient patients eventually become platinum resistant as well [14].

## 2. Epigenetic Modifications

Epigenetic modifications are mechanisms that alter the expression of a gene but do not change the DNA sequence itself [15,16]. These epigenetic modifications act together to regulate normal functioning of the genome, with abnormal epigenetic regulation often resulting in the development of specific disease states such as cancer [15,16]. Key epigenetic regulators include DNA methylation, histone modifications, and microRNAs (miRNAs).

**Figure 1:**
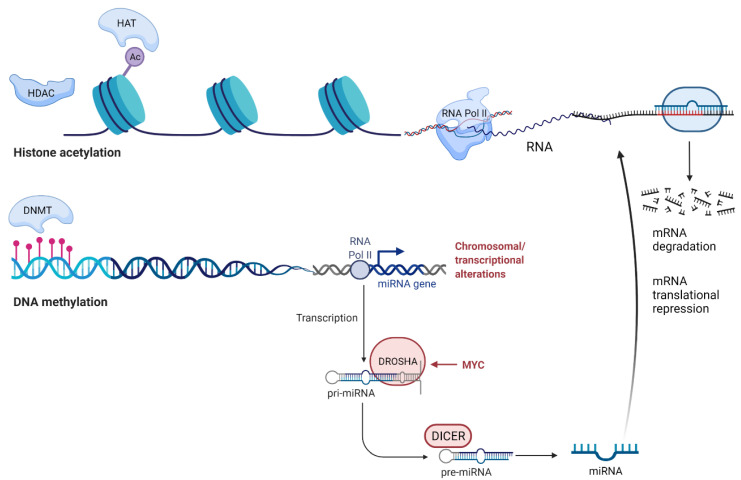
This diagram summarises the complexity of epigenetic modifications. DNA methylation, histone modification (histone acetylation in this diagram), and miRNA expression influence the epigenetics of ovarian cancer development and progression towards treatment resistance. Hypermethylation of gene promoters is associated with suppression of gene expression, a process catalysed by DNA methyl transferase (DNMT) enzymes. HAT enzymes add acetyl groups to the histone surface, which increases the accessibility of RNA polymerase II, leading to gene expression. HDAC enzymes remove the acetyl groups from histones and restrict access by RNA polymerase, resulting in decreased gene expression. miRNAs target mRNAs by binding with their 3′-UTR, leading to mRNA degradation or translational repression. Figure adapted from “Cancer Epigenetics” and “miRNA in Cancer”, by BioRender.com (2021). Retrieved from https://app.biorender.com/biorender-templates (accessed on 4 Novermber 2021).

The modifications that will be discussed in this review are DNA methylation, histone acetylation, and microRNA (miRNA) as epigenetic modulators, as they are most commonly studied in serous ovarian cancer (summarised in Figure 1). This review focused on studies that were performed in patient cohorts that included histologically confirmed serous ovarian cancer, xenograft models, epithelial or serous ovarian cancer cell lines (KURAMOCHI, OVSAHO, SNU119, C0V362, OVCAR4, COV318, JHOS4, TYKNU, OVKATE, CAOV4, OAW28, and JHOS2), as characterised by Domcke et al. [17]. Studies using OVCAR3 and CAOV3 were included as these cell line possess *TP53* mutations and substantial copy-number changes, key characteristics of HGSOC.

Epigenetic mechanisms have one thing in common: they modulate the expression of genes that are involved in the development, progression, and chemoresistance of ovarian cancer. This review will focus on the roles of epigenetic modifications in the development and progression to chemoresistance in HGSOC.

### 2.1. DNA Methylation

DNA methylation is a key epigenetic regulator of gene expression in which DNA methyltransferase (DNMT) enzymes catalyse the addition of a methyl group onto the fifth carbon of a cytosine ring to form methyl cytosine [18,19]. DNA methylation occurs mostly on cytosines which are followed by a guanine in CpG dinucleotides. Stretches of CpG-rich DNA, known as CpG islands, are often located in the regulatory region of genes [16,20]. Increased methylation of cytosines located in CpG islands within the promoter region of a gene is known as hypermethylation and causes binding of proteins to the methylated cytosines within the DNA strand [19]. The cytosine-bound proteins inhibit the ability of transcription factors and RNA polymerase to bind to DNA and undergo transcription, resulting in decreased gene expression. In contrast, decreased methylation of CpG sites within promoter regions, known as hypomethylation, results in increased gene expression. Abnormal methylation patterns are common in cancer and can typically be characterised by global hypomethylation and gene-specific hypermethylation of tumour suppressor genes [20,21].

### 2.2. Histone Modification

There are several known histone modifications, such as acetylation, phosphorylation, and methylation. The most comprehensively studied modification in ovarian cancer, not specifically HGSOC, is histone acetylation.

DNA is coiled around and octomer of four histone proteins (H2A, H2B, H3, and H4) [22,23], resulting in compact chromatins, which restrict the access of transcription factors to the DNA. Each of these histone proteins contains a side chain dense with lysine and arginine residues. These side chains are subject to posttranslational modifications, which involve the addition or removal of chemical groups, such as acetyl (histone acetylation) or methyl (histone methylation) groups. Histone acetylation is a histone modification wherein an acetyl group is added to lysine residues. Histone acetyl transferase (HAT) enzymes add acetyl groups to the lysine residues on the histone surface, which increases the accessibility of RNA polymerase II, leading to gene expression. Histone deacetylase (HDAC) enzymes remove the acetyl groups from histones and restore the compact chromatin structure by increasing the electrostatic interactions between the histones and DNA, subsequently restricting access by RNA polymerase and resulting in decreased gene expression.

HDACs are divided into four classes: class I (HDACs 1, 2, 3, and 8) is found in the nucleus and is the most prevalent, whereas class II (HDACs 4, 5, 6, 7, 9, and 10) and class IV (HDAC11) are found in both the nucleus and cytoplasm, and all are zinc-dependent and considered classical HDACs. Class III (SIRT1, SIRT2, SIRT3, SIRT4, SIRT5, SIRT6, and SIRT7) is nicotinamide adenine dinucleotide (NAD)-dependent and found in both the nucleus and cytoplasm [24]. HDACs are aberrantly expressed in cancer, including ovarian cancer in general, but studies specific to HGSOC have not been reported [25,26,27].

### 2.3. MicroRNAs

MicroRNAs, or miRNAs, are small, highly conserved single-stranded non-coding RNAs (19–25 nucleotides) that are involved in post-translational regulation of gene expression. miRNAs negatively regulate target protein-coding genes through binding with the 3′-UTR (untranslated region), which causes messenger RNA (mRNA) degradation or translational repression [28,29]. miRNAs are transcribed in the nucleus by RNA polymerase II enzyme into double-stranded precursors known as primary miRNA transcripts (pri-miRNAs), which are then processed by Drosha and DGCR8 to produce premature miRNAs (pre-miRNAs). The pre-miRNAs are translocated to the cytoplasm and cleaved by the Dicer complex into mature miRNAs. The mature miRNAs, together with the ribonucleoprotein complex, form the RNA-induced silencing complex (RISC), which directs the complex to bind to the target mRNA. miRNAs use between seven and eight nucleotides from their 5′end to target the 3′-UTR of the mRNA to inhibit translation or induce mRNA degradation. As binding of miRNA to mRNA does not require complete base-pair complementarity, each miRNA may be able to regulate the expression of several hundred genes and, conversely, one mRNA can be regulated by multiple miRNAs [30].

miRNAs can act as oncogenic miRNAs (oncomiRs) by targeting mRNA that encode for tumour-suppressor proteins, and as tumour-suppressor miRNAs by targeting mRNA that encode for oncogenic proteins. miRNA expression is typically altered in cancers, with tumour-suppressor miRNA subsets typically downregulated and oncogenic miRNAs upregulated [28,31]. Dysregulation of miRNAs can also occur in cancer from other aberrant epigenetic patterns, including abnormal DNA methylation or histone modifications [28].

## 3. Epigenetic Changes and Their Role in HGSOC Chemoresistance

HGSOC is highly controlled by epigenetic modifications. DNA methylation and histone modification have both been extensively studied and targeted with new treatment regimens to attempt to overcome platinum chemoresistance.

### 3.1. DNA Methylation Changes in HGSOC Chemoresistance

The potential role of altered DNA methylation patterns in the acquisition of chemoresistance in HGSOC at both the genome-wide and single-gene level has been extensively studied (summarised in Table 1 and Table 2).

One of the first studies of genome-wide methylation patterns in chemoresistant HGSOC found 749 differentially methylated probes (DMPs) between chemoresistant and chemosensitive tumour samples, which were associated with 296 genes that were both differentially methylated and differentially expressed in chemoresistant samples [32]. Interestingly, approximately 60% of these DMPs were hypermethylated in the chemoresistant samples, as opposed to the typical pattern of global hypomethylation in cancer [32]. Similar results were seen in further studies into genome-wide methylation patterns in chemoresistant HGSOC, with Chan et al. showing that platinum resistant HGSOC patient samples had significantly increased levels of global hypermethylation when compared to platinum sensitive samples, finding 5844 DMPs between platinum-sensitive and -resistant HGSOC samples [33]. Cardenas et al. also found 452 genes that were hypermethylated specifically in recurrent platinum-resistant HGSOC tumour samples [34]. However, whole-genome analysis of methylation patterns performed by Lund et al. found that 84% of the 1488 differentially methylated sites between cisplatin-sensitive and -resistant HGSOC cell lines were hypomethylated in the chemoresistant cells [35]. Additionally, 64 of the 109 differentially expressed genes found by Wu et al. were upregulated in chemoresistant HGSOC patient samples, indicating hypomethylation of these genes [36]. Further studies are therefore required to determine the role global DNA methylation patterns play in the development of chemoresistance in HGSOC.

Investigations into the role of DNA methylation in HGSOC chemoresistance have also focused on differential methylation of single genes, especially in genes associated with EMT. Biological pathway analysis of the 452 hypermethylated genes discovered by Cardenas et al. found that EMT was one of the most highly enriched biological pathways in chemoresistant HGSOC [34], indicating that aberrant methylation of genes within the EMT pathway plays a role in the development of chemoresistance in HGSOC. CpGs within *MSX1* were found to have decreased methylation levels in tumour samples from chemoresistant HGSOC patients [37]. *MSX1* is crucial in controlling epithelial–mesenchymal interactions during embryogenesis [37,38], and DNA methylation of these CpGs is thought to impair MSX1-dependent mesenchymal embryogenesis [39], suggesting that hypomethylation of *MSX1* plays a role in the occurrence of EMT through promoting the transition of cancer cells to a mesenchymal phenotype. *LAMA3*, an integral part of the cell basement membrane, was also found to be hypermethylated in chemoresistant HGSOC tissue samples [40]. Reduced *LAMA3* expression has previously been linked to both loss of the basement membrane and EMT [41], indicating that *LAMA3* hypermethylation may also be involved in the development of EMT. Lum et al. also identified several differentially methylated genes in chemoresistant HGSOC involved in EMT, including *SOX9*, *ZIC1*, and *TWIST* [32], further implicating differential methylation patterns of EMT genes to the development of chemoresistance in HGSOC.

The wingless/integrated (Wnt) signalling pathway is highly associated with EMT as well as CSCs and has been implicated in HGSOC chemoresistance due to abnormal methylation of genes within the pathway. Activation of the Wnt pathway occurs through interaction of Wnt proteins with the frizzled (FZD) family of transmembrane receptors, triggering phosphorylation of downstream proteins [42,43]. Two different FZD receptors, *FZD1* [32] and *FZD10* [44], have both been found to be differentially methylated in chemoresistant HGSOC tumour samples. *GSK3B*, another Wnt-associated gene, also has altered methylation patterns in chemoresistant and chemosensitive HGSOC tissue samples [32].

While there has not been as much evidence on differential methylation of DNA repair genes being involved in HGSOC chemoresistance, there has been one reported case of hypomethylation of *BRCA1* in a relapsed HGSOC tumour sample in comparison to the patient’s primary tumour [45]. Global methylation patterns of both samples indicated that methylation patterns were not altered genome wide and that this change was gene specific, suggesting a potential role in aberrant methylation of *BRCA1* in the acquisition of chemoresistance in HGSOC.

Altered methylation patterns of tumour suppressor genes have also been implicated in chemoresistance development in HGSOC. *AKAP12* is a scaffolding protein previously found to be a tumour suppressor gene [46]. Protein and transcript expression of *AKAP12* was increased in paclitaxel resistant HGSOC cell lines, which was associated with low levels of *AKAP12* gene methylation [47]. Interestingly, mRNA transcript expression of *AKAP12* was found to increase in cells after induced EMT [48], indicating another potential mechanism of chemoresistance of *AKAP12* hypomethylation. *BLU* and *ZNF671*, two genes which are thought to act as tumour suppressors in other cancer types [49,50], have significantly higher methylation in chemoresistant HGSOC tumour samples [51,52]. *DOK2*, a known lung cancer tumour suppressor, is also differentially methylated in chemoresistant samples [32], further implicating altered methylation patterns of tumour suppressor genes in developing resistance to chemotherapy in HGSOC.

**Table 1:** Genome-wide methylation studies of chemoresistance in HGSOC.

Author	Differentially Methylated Genes	Methylation Level in Chemoresistant HGSOC	Tissue Source	Reference
Cardenas et al. 2020	*ADAM10*, *AGT*, *AKT2*, *BDNF*, *CCL14*, *CRMP1*, *CSF3*, *CUL7*, *FGF7*, *FGF10*, *FOXA2*, *FSTL1*, *GAB2*, *NDRG2*, *NKX2-1*, *NR1H4*, *RASSF1*, *TGFBR1*	Hypermethylated	Tumour samples, *n* = 73	[34]
Chan et al. 2021	*OR51L1*, *OR51I1*, *OR51F1*, *OR51B6*, *HBBP1*, *TMEM200A*, *DLG2*	Hypermethylated	Tumour samples, *n* = 30	[33]
Lum et al. 2013	*GSK3B*, *DOK2*, *APRT*, *OXSR1*, *CENPB*, *FZD1*, *ESRRA*, *HIRIP3*, *GTF2B*, *SGPL1*, *GABPA*, *TWIST1*, *MDH1*, *NR2E1*, *NR3C2*, *SOX9*, *TOB1*, *UNG*, *ZIC1*	Differentially methylated	Tumour samples, *n* = 36	[32]
Lund et al. 2017	*AQP3*, *CTSB*, *CYP24A1*, *PRSS56*, *ECEL1*, *SPOCK1*, *SYNE1*, *PBX1*, *PTGDS*, *ST3GAL5*, *FOSL1*, *IL8/CXCL8*, *ARRDC4*, *TNFAIP3*, *ODC1*, *RNF43*, *HERC5*, *OASL*, *KLF4*, *IL6*	Hypermethylated	Primary cell lines derived from patients (M019i, OC002) and cisplatin-resistant clones (M019iCi, OC002Ci)	[35]
Wu et al. 2020	*KIT*, *FOXM1*, *FGF2*, *HIST1H4D*, *ZFPM2*, *IFIT2*, *CCNO*, *MGP*, *RHOBTB3*, *CDC7*	Differentially methylated	DNA methylation data from patients, *n* = 28	[36]

**Table 2:** Single-gene methylation studies of chemoresistance in HGSOC.

Author	Gene	Methylation Level in Chemoresistant HGSOC	Tissue Source	Reference
Bateman et al. 2015	*AKAP12*	Hypomethylated	OV90 and paclitaxel-resistant OV90-TR1, E3 cell line from chemoresistant patient	[47]
Bonito et al. 2016	*MSX1*	Hypomethylated	Tumour samples, *n* = 78	[37]
Chiang et al. 2013	*BLU*	Hypermethylated	Tumour samples, *n* = 40	[51]
Feng et al. 2021	*LAMA3*, *NCALD*	Hypermethylated	Tumour samples, *n* = 61	[40]
Li et al. 2021	*MGRN1*	Hypermethylated	Tumour samples, *n* = 96	[53]
Mase et al. 2019	*ZNF671*	Hypermethylated	DNA methylation data from patients, *n* = 584	[52]
Sharma et al. 2019	*POTEC*, *POTEE*, *POTEF*	Hypomethylation	DNA methylation data from patients, *n* = 10	[54]
Tomar et al. 2016	*CSK*	Hypermethylated	DNA methylation data from patients, *n* = 91	[55]
Tomar et al. 2017	*FZD10*	Hypomethylated	Tumour samples, *n* = 18	[44]

### 3.2. Histone Modifications in HGSOC Chemoresistance

The role of post-translation histone modifications in HGSOC chemoresistance is still relatively understudied. Histone hypoacetylation by HDACs, and subsequent gene repression, has been associated with tumorigenesis by upregulating genes involved in cell proliferation and migration, and downregulating genes involved in cell differentiation and apoptosis [27,56].

In general, ovarian cancer chemoresistance is often associated with increased HDAC activity. Class I HDACs 1, 2, and 3 were highly expressed in a large proportion of HGSOC (64%) and were associated with highly proliferating tumours, as determined by Ki-67 labelling [57]. The overexpression of these three HDACs is also associated with the development of platinum resistance in ovarian cancer [58].

The overexpression of HDAC1 in the nucleus was significantly associated with decreased progression-free survival (PFS) and overall survival (OS) in serous ovarian cancer [26]. The expression of both HDAC1 and HDAC7 increased after chemotherapy, and they have been shown to be overexpressed in ovarian cancer stem cells [59]. HDAC1 and HDAC7 maintain the cancer stem cell phenotype, are associated with metastasis and tumour relapse, and can be inhibited by vorinostat, an approved HDAC inhibitor [60]. Therefore, these HDACs play a potential role in conferring chemoresistance in HGSOC and are potential targets for therapeutic inhibition.

In a recent study by Ali et al. low expression of HDAC6 was shown to be associated with decreased OS in HGSOC patients [61]. However, in the same study, patient-derived HGSOC cell lines with high HDAC6 expression were shown to be more metastatic and have higher cell proliferation than those with low HDAC6 expression. The siRNA knockdown of HDAC6 and pharmacological inhibition by HDAC6 inhibitor decreased cell proliferation and migration [61]. High expression of HDAC6 was also more recently shown to be associated with decrease in PFS and OS [62]. These results suggest that inhibition of HDAC6-specific inhibition may be a potential therapeutic strategy in HGSOC.

### 3.3. miRNA in HGSOC Chemoresistance

The role of miRNAs in chemoresistance is the regulation of genes involved in apoptosis, proliferation, regulation of cell cycle, and DNA repair, all of which are pathways targeted or exploited by chemotherapeutic agents. miRNAs have been shown to both increase sensitivity and promote resistance to platinum chemotherapy. However, those that increase platinum chemoresistance have been compiled in this review (summarised in Table 3).

An analysis of the TCGA dataset [12] by Nishimura et al. found that miR-520d-3p (also called miR-520d) is associated with increased survival in patients with serous ovarian cancer [63]. One of the targets of miR-520d-3p is the oncogene *EphA2* (EPH receptor A2). High expression of EphA2 is significantly associated with poor 5-year OS in patients with HGSOC [64]. The expression of miR-181a led to decreased levels of *RB1*, a protein that controls cells division and protects cells from genomic instability in fallopian tube cells. Simultaneously, miR-181a also inhibited the expression of *STING*, allowing the genomically unstable cells to be protected from being destroyed by interferon-mediated cell death [65].

miR-484, miR-642, and miR-217 were downregulated in tumours that were non-responsive (stable or progressive disease) to platinum and taxane combination [66]. Additionally, miR-484 was shown to confer chemosensitivity to combined platinum and taxane chemotherapy in vivo. Increased miR-484 expression was associated with a lower expression of *VEGFB* and *VEGFR2* in tumours that were responsive to treatment, suggesting that miR-484 exerts its effects through the regulation of angiogenic factors that control the formation of new vasculature [66].

The miR-200 family, consisting of miR-200a, miR-200b, miR-200c, miR-141, and miR-429, has been extensively studied in ovarian cancer chemoresistance. Nam et al. showed that the high expression of miR-141 and miR-200c is associated with platinum chemoresistance in ovarian cancer [67]. However, a later study, and further subsequent studies, showed that a high expression of miR-200c is associated with a better clinical response, and a low expression of miR-200c is associated with recurrence [68].

The inhibition of miR-141 and miR-200c is involved in resistance to platinum- and taxane-based chemotherapies by triggering EMT. The re-expression of the miR-200 family reverts the EMT phenotype by inducing mesenchymal-to-epithelial transition (MET), and resensitizes ovarian cancer cells to platinum- and taxane-based chemotherapies [69].

Low expression of miR-200c results in the aberrant expression of *ZEB1* and repression of E-cadherin, but the re-expression of miR-200c restores E-cadherin and reduces cell migration and invasion [68]. miR-200c also directly targets class IIIβ-tubulin (*TUBB3*), which encodes a tubulin isotype known to mediate chemoresistance [70]. Restoration of miR-200c results in sensitivity to microtubule-targeting chemotherapy agents, such as paclitaxel [71]. Additional studies showed that miR-200c was downregulated in ovarian cancer cell lines and advanced stage serous ovarian tumours and restoration of miR-200c in vivo targets *TUBB3* and reduces tumour burden by increasing sensitivity to taxanes [72].

These studies show that miRNAs can affect the response to standard platinum- and taxane-based chemotherapies used to treat HGSOC, by targeting multiple cellular pathways including the EMT/MET pathways and microtubule assembly. miRNAs can also target the angiogenesis pathway, which may affect sensitivity anti-angiogenic therapies that are used in recurrent HGSOC.

The inhibition of DNA damage repair proteins by miRNAs also plays a role in resistance to DNA-damaging chemotherapy in HGSOC treatment. miR-9 downregulates BRCA1 expression by directly binding to the 3′-UTR of *BRCA1*. In serous ovarian cancer, higher levels of miR-9 were associated with decreased BRCA1 expression and showed increased response to platinum chemotherapy and longer PFS [73]. This suggests that miR-9 mediates the downregulation of BRCA1, which is involved in the repair of DNA damage, thus increasing sensitivity of ovarian cancer to DNA-damaging chemotherapy.

miR-93 was upregulated in platinum-resistant HGSOC cells lines. miR-93 was shown to downregulate PTEN expression in HGSOC cells by, similar to the above, directly binding to the 3′-UTR of *PTEN*. The suppression of miR-93 by miR-93 antisense oligonucleotides increased PTEN expression and apoptotic activity in the ovarian cancer cells, suggesting that miR-93 may play a role in regulating platinum sensitivity [74].

Several studies comparing platinum-sensitive and platinum-resistant HGSOC patient cohorts have also identified miRNAs that are potential biomarkers of platinum chemoresistance. miR-206 was highly expressed in primary platinum-resistant (majority serous) ovarian cancer patients (classified by incomplete response to primary therapy) [75]. This was achieved by downregulating the expression of *Cx43* [75], a gap junction protein that promotes cisplatin cytotoxicity [76]. An analysis of the International Cancer Genome Consortium (ICGC) data [45] by Qi et al. found that miRNA-454-3p, miRNA-98-5p, miR-183-5p, and miR-22-3p may be biomarkers for predicting platinum resistance in HGSOC, as they are associated with PFS and OS [77]. The target genes of these four miRNAs are implicated in cancer progression-related processes, such as transcriptional regulation, morphogenesis, and cell migration and proliferation [77]. The target genes were also shown to be enriched in platinum resistance-associated pathways, including Wnt/β-catenin signaling (cell proliferation and apoptosis), ATM signaling (DNA damage and repair), SAPK/JNK (apoptosis), RhoGDI signaling (tumour proliferation and metastasis), and CDK5 signaling (cell cycle) [77].

**Table 3:** miRNA studies of chemoresistance in HGSOC.

Author	miRNA Expression	Affected Genes	Expression in Chemoresistant HGSOC	Reference
Fu et al. 2012	miR-93	*PTEN*	miR-93 downregulates PTEN expression by direct binding to the 3′-UTR of PTEN.	[74]
Knarr et al. 2020	miR-181a	*RB1*	High expression of miR-181a downregulates RB1 expression.	[65]
Leskela et al. 2011	miR-200c	*ZEB1*, *E-cadherin*, and *TUBB3*	Low expression of miR-200c downregulates ZEB1 and E-cadherin. Increased expression of miR-200c downregulates TUBB3 expression.	[68]
Nam et al. 2008	miR-141	EMT pathway	High expression of miR-141 is associated with platinum chemoresistance.	[67]
Nishimura et al. 2013	miR-520d-3p (miR-520d)	*EphA2*	High expression of EphA2 is significantly associated with poor 5-year OS in HGSOC patients.	[63]
Sun et al. 2013	miR-9	*BRCA1*	miR-9 downregulates BRCA1 expression by direct binding to the 3′-UTR of BRCA1.	[73]
Vecchione et al. 2013	miR-484, miR-642, and miR-217	*VEGFB* and *VEGFR2*	miR-484, miR-642, and miR-217 are downregulated in tumours that were non-responsive to platinum and taxane.	[66]
Yu et al. 2020	miR-206	*Cx43*	High expression of miR-206 downregulates Cx43 expression and is associated with platinum chemoresistance.	[75]

## 4. Current Treatment with Epigenetic Modifiers Targeting Chemoresistance in HGSOC

The majority of patients with advanced high-grade serous ovarian cancer (HGSOC) develop recurrent disease within 3 years and succumb to the disease within 5 years [78]. Although initial recurrences are usually platinum sensitive, patients eventually develop resistance to platinum-based chemotherapy [78]. Accordingly, one of the major problems in the treatment of HGSOC and disease recurrence is the development of chemotherapy resistance [79].

### 4.1. DNA Methyltransferase Inhibitors (DNMTi)

The reversibility of epigenetic modifications makes them a potential treatment strategy to enhance response to chemotherapy in chemoresistant HGSOC. One promising area of epigenetic treatments is treatments that reduce DNA methylation by inhibiting the ability of DNMT enzymes. These DNMT inhibitors (DNMTi) are cytosine analogues which are incorporated into the DNA strand during replication and covalently bind to DNMTs, making them inactive. As a result of this decrease in DNMT activity, CpG sites which were previously methylated become unmethylated during cell replication, and transcription of genes previously silenced due to promoter hypermethylation is increased [80,81]. Two DNMTis, azacitidine (5-azacitidine) and decitabine (5-aza-2′-deozycitidine), are approved for use in treating myelodysplastic syndrome [6,80], with a second-generation DNMTi, guadecitabine, currently being tested in clinical trials [82,83,84].

While azacitidine and decitabine have not shown any effectiveness in treating solid tumours as single agents, preclinical studies have found that combination therapy of a DNMT inhibitor alongside chemotherapy increased the sensitivity to platinum in platinum-resistant ovarian cancer cell lines [85,86]. Pre-treatment with azacitidine or decitabine before platinum chemotherapy in platinum-resistant patients resulted in an objective response rate (ORR) of more than 20% [87,88].

This combination therapy has therefore been the focus of clinical trials investigating DNMT inhibitors in chemoresistant HGSOC (Table 4). Fang et al. first assessed a combination of repeated low-dose decitabine and platinum chemotherapy to improve sensitivity to platinum therapy in a phase 1 trial [89]. The combination was effective, with one patient of the ten enrolled developing a complete response and three more developing stable disease; adverse effects were minimal. Importantly, the combination was found to improve sensitivity to carboplatin and reduce DNA methylation, with the methylation rates of the ovarian-cancer-associated genes *HOXA11* and *BRCA1* reduced after 8 days [89]. This combination therapy was studied further in a phase 2 trial conducted by Matei et al. which saw an ORR of 35% and PFS of 10.2 months after a combination of low-dose decitabine and carboplatin [88]. Of the 17 patients enrolled, one achieved a complete response, five had a partial response, and six developed stable disease after combination treatment. Additionally, both global and gene-specific DNA methylation levels were reduced in tumours, with higher rates of demethylation seen in patients with greater PFS. Demethylated genes were involved in biological pathways including apoptosis and Wnt signalling, and ovarian-cancer-associated genes *MLH1*, *RASSF1A*, *HOXA10*, and *HOXA11* all had reduced methylation after combination treatment [88]. These results contrasted with a phase 2 trial by Glasspool et al. which found no clinical response in chemoresistant HGSOC patients receiving combination decitabine and carboplatin [90]. Severe adverse effects of neutropenia and hypersensitivity were also seen after combination treatment, resulting in earlier closure of the trial. However, this study tested a higher dose of decitabine than the previous studies [88,89] and did not test repeated decitabine dosing, only delivering decitabine on day 1 of the treatment cycle. The study authors noted that these differing results may be due to the 5-day decitabine schedule used in the other trials, which would allow for prolonged demethylation during each cycle of treatment and would increase response to chemotherapy [90]. Additionally, high doses of DNMTis have been found to be cytotoxic [91,92], indicating that repeated low-dose decitabine may be more clinically relevant and effective for inducing hypomethylation.

Combination therapy of azacitidine and carboplatin has also been assessed in a phase 1b-2a clinical trial by Fu et al. wherein 1 out of 29 patients enrolled achieved a complete response, 3 achieved partial responses, and 10 developed stable disease (ORR = 13.8%, PFS = 3.7 months), with no toxicities observed [87]. Platinum-resistant patients were found to have better outcomes with the combination, with these patients achieving an ORR of 22% and PFS of 5.6 months [87].

Clinical trials testing carboplatin in combination with the second-generation DNMTi guadecitabine have also recently been conducted. A phase 1 study conducted by Matei et al. [93] tested guadecitabine in the 5-day dosing followed by carboplatin on day 8, similar to treatment schedules found effective in previous trials [88,89]. The combination achieved partial responses in 3/20 patients enrolled (ORR = 15%), with a further six developing stable disease. The average demethylation of the DNA-repetitive element LINE1 was 19% at day 8, and decreased levels of LINE1 methylation after cycle 1 were maintained or decreased during subsequent cycles [93], providing evidence of hypomethylating effects of the guadecitabine and carboplatin combination. This combination was further studied in a phase 2 trial conducted by Oza et al. which did not find any statistically significant differences in PFS between the combination therapy (PFS = 16.2 weeks) and control treatment (treatment of choice, PFS = 9.1 weeks) [94]. However, the 6-month PFS rate was significantly higher in patients treated with the combination (37%) compared to control treatments (11%), with the authors noting that these results suggest that a subgroup of HGSOC patients may benefit from the combination therapy.

**Table 4:** Clinical trials of DNMTis in chemoresistant HGSOC.

Authors	Drugs	Study Design	Dosage	Clinical Response	Other Results	Reference
Fang et al. 2010	Decitabine + carboplatin	Phase 1 (*n* = 10)	Decitabine: 10 or 20 mg/m^2^ i.v. days 1–5 of 28-day cycle Carboplatin: i.v. day 8	1 CR 3 SD	Minimal adverse effects (commonly Grade 1–2). Global and gene-specific demethylation in PBMCs and tumours	[89]
Matei et al. 2012	Decitabine + carboplatin	Phase 2 (*n* = 17)	Decitabine: 10 mg/m^2^ i.v. days 1–5 of 28-day cycle Carboplatin: i.v. day 8	1 CR 5 PR 6 SD	ORR: 35% PFS: 10.2 months	[88]
Glasspool et al. 2014	Decitabine + carboplatin	Phase 2 (*n* = 29)	Decitabine: 90 and subsequently 45 mg/m^2^ i.v. day 1 of 28-day cycle Carboplatin: i.v. day 8	3 PR 5 SD	Trial terminated due to lack of clinical effect and severe adverse effects (hypersensitivity, neutropenia)	[90]
Fu et al. 2011	Azacitidine + carboplatin	Phase 1b–2a (*n* = 29)	Azacitidine: 75 mg/m^2^ s.c. days 1–5 of 28-day cycle Carboplatin: i.v. day 2	1 CR 3 PR 10 SD	ORR: 13.8% (22% in platinum-resistant patients) PFS: 3.7 months (5.6 months in platinum-resistant patients)	[87]
Matei et al. 2018	Guadecitabine + carboplatin	Phase 1 (*n* = 20)	Guadecitabine: dose escalation (45 to 60 mg/m^2^) s.c. days 1 of 28-day cycle Carboplatin: i.v. day 8	3 PR 6 SD	ORR: 15% PFS: 3.7 months Minimal adverse effects (commonly Grade 1–2) Demethylating effects observed and maintained over subsequent treatment cycles	[93]
Oza et al. 2020	Guadecitabine + carboplatin	Phase 2 (*n* = 100)	Guadecitabine: 30 mg/m^2^ s.c. days 1 of 28-day cycle Carboplatin: i.v. day 8	21 responders (CR + PR)	ORR: 16% No difference in median PFS 6-month PFS rate: 37%	[94]

i.v.—intravenous; s.c.—subcutaneous; CR—complete response; PR—partial response; SD—stable disease.

To date, DNMTis are the epigenetic modulators that have progressed the furthest in clinical trials, with next-generation DNMTis that confer less severe and dose-limiting side effects being investigated [81].

### 4.2. Histone Deacetylase Inhibitors (HDACi)

The mechanism of action of HDAC inhibitors (HDACi) is via the alteration of gene transcription, affecting proteins involved in cell growth, the promotion of cell differentiation, and apoptosis [95]. HDACis act by targeting the zinc ion and inhibiting the catalytic function of class I, II, or IV HDACs, and are classified based on their specificity; pan- or class-specific-HDACis. Pan-HDACis vorinostat, belinostat, and panobinostat, and class I-specific HDACi romidepsin has been FDA approved for the treatment of haematological malignancies [80]. Class III HDACs, which are not zinc-dependent, are not inhibited by currently approved or available HDACis [96] and will not be discussed in this review.

In vitro studies showed that HDACis (pan and class-specific) sensitize HGSOC cells to DNA-damaging drugs, such as platinum chemotherapy, by increasing apoptosis-mediated cell death caused by platinum chemotherapy treatment [97,98]. The analysis of gene expression after combined treatment with HDACis and cisplatin showed upregulated expression of pro-apoptosis genes APAF1 (apoptotic protease activating factor), PUMA (p53 upregulated modulator of apoptosis), BAK1 (Bcl-2 homologous antagonist killer), and downregulation of anti-apoptotic gene BIRC5 (baculoviral inhibitor of apoptosis repeat-containing 5; survivin), compared to treatment with HDAC inhibitors or cisplatin alone [97].

Panobinostat downregulated genes of the cyclin E and homologous recombination repair pathways, and synergistically with the PARP inhibitor olaparib, reduce the cell viability and growth in homologous recombination-proficient ovarian cancer cells and xenografts [99]. Similarly, entinostat was shown to inhibit homologous recombination repair by reducing BRCA1 expression and stalling replication fork progression, leading to irreparable DNA damage and subsequent cell death [100]. These studies suggest a potential use for HDACis to enhance the activity of PARP inhibitors.

Several treatment combinations that include HDACis have been trialled to determine whether HDACis can resensitize platinum-resistant HGSOC to platinum chemotherapy (Table 5). However, the use of single-agent HDACis to increase anti-tumour activity in platinum-resistant ovarian cancer has not shown much success due to their limited therapeutic effects and high toxicity [101].

A phase 1 dose-escalating clinical trial of HDACi vorinostat and chemotherapy agents carboplatin and gemcitabine in platinum-sensitive ovarian cancer (including HGSOC) with a first recurrence was terminated early due to haematological toxicity [102]. Of the s (out of fifteen) patients that were evaluable, six had partial response and one had stable disease [102].

Belinostat is better tolerated than vorinostat in combination treatments regimens. In a phase 2 clinical trial evaluating the efficacy of combined belinostat and carboplatin, with 27 patients, the majority of which had HGSOC, the ORR was 7.4% (one complete response and one partial response, 5% CI, 0.9–24.3%). The study was closed due to the lack of drug activity and concluded that the addition of belinostat did not resensitize platinum-resistant ovarian cancer to carboplatin [103].

**Table 5:** Clinical trials of HDACis in chemoresistant HGSOC.

Authors	Drugs	Study Design	Dosage	Clinical Response	Other Results	Reference
Dizon et al. 2012	Belinostat + carboplatin	Phase 2 (*n* = 27)	Belinostat: 1000 mg/m^2^ i.v. days 1–5 of 21-day cycle Carboplatin: i.v. cycle day 3	1 CR 1 PR 12 SD	ORR: 7.4% Grade 3–4 adverse events noted: neutropenia, thrombocytopenia, vomiting, anemia, allergic reaction, nausea. Trial terminated due to lack of clinical effect.	[103]
Matulonis et al. 2015	Vorinostat + carboplatin + gemcitabine	Phase 1 (*n* = 15)	Vorinostat: dose escalation (200–400 mg) once or twice daily, days 1/2/1+2 of 21-day cycle Carboplatin: i.v. cycle day 1 or 2 Gemcitabine: 1000 mg/m^2^ i.v. cycle day 8	1 SD 6 PR	ORR: 40% Grade 3–4 adverse events noted: neutropenia, thrombocytopenia. Trial terminated due to lack of clinical effect.	[102]

i.v.—intravenous; CR—complete response; PR—partial response; SD—stable disease.

To date, there have not been any clinical trials utilising panobinostat or romidepsin in combination with standard-of-care therapy for chemoresistant HGSOC. There is evidence that HDACis could potentially play a role in the treatment of ovarian cancer, but they would need to be further improved to increase their efficacy whilst having tolerable side effects.

### 4.3. Combination of DNMT and HDAC Inhibitors

Stone et al. have previously showed that epigenetic modulating drugs, DNMT inhibitors and HDAC6 inhibitors, individually increase immune signalling in HGSOC cell lines [104]. DNMT inhibitors upregulate immune signalling in ovarian cancer, including interferon response, tumour-associated antigens, and antigen presentation [105]. Follow-up studies showed that the combination of DNMT and HDAC inhibitors upregulated type I interferon response, which led to an increased expression of cytokines and the MHC I antigen presentation complex in HGSOC cell lines [106]. In an in vivo model ovarian cancer, DNMT inhibitor alone and the combination of DNMT and HDAC6 inhibitors decreased the tumour burden and increased survival, with the epigenetic therapy combination also exhibiting a trend towards an immunogenic tumour microenvironment [106].

### 4.4. miRNA Inhibition/Replacement Therapy

Therapeutic approaches to regulate the expression of miRNAs include miRNA replacement by miRNA mimics or miRNA inhibition by antimiRs. As discussed above, each miRNA can potentially target hundreds of mRNAs as they do not require perfect binding complementarity. This can lead to potential off-target effects in miRNA-targeting therapy. Another limitation of miRNA-targeting therapy is the short half-life of miRNA mimics and antimiRs, which are also almost immediately degraded by nucleases and therefore require a safe delivery system [107]. These issues add complexity to the application of miRNA therapy. Most studies to date of miRNA mimic and antimiR therapy have focused on reducing the tumour burden and preventing disease metastasis in ovarian cancer, rather than targeting chemoresistance.

Many preclinical studies of miRNA-targeting therapies have not yet developed into clinical trials. As recently reviewed by Zhang et al. the ten miRNA-targeting drugs that have been in clinical trials have hundreds of unapproved targets, compared to approved drugs that have no more than five unapproved targets [108].

Previous studies (not in cell lines listed above) showed that miR-182 is involved in chemoresistance of ovarian cancer by downregulating the cell cycle gene *PDCD4*, and that the suppression of miR-182 increases platinum- and taxane-induced apoptosis [109,110]. Treatment of the OVCAR3 cell line with anti-miR-182 significantly reduced cell proliferation and tumour invasion [111]. The combination of cisplatin and anti-miR-182 further inhibited cell proliferation [111]. In mice with OVCAR3 xenografts, anti-miR-182 treatment also reduced tumour growth rate and tumour size and restored the expression of several cell cycle genes, including *PDCD4* [111].

An analysis of the TCGA database by Dwivedi et al. showed that lower expression of miR-15a and miR-16, and subsequent upregulation of their target *BMI1*, a regulator of CSCs [112], is associated with decreased OS in HGSOC patients [113]. The EMT pathway, which is implicated in ovarian cancer chemoresistance, can also be inhibited by miR-15a and miR-16 [113]. Treatment with miR-15a and miR-16 improved the response to cisplatin in HGSOC cell lines and a preclinical chemoresistant ovarian cancer mouse model and resulted in a decreased expression of BMI1 [113]. The combination of miR-15a and miR-16 is more effective in reducing the tumour burden compared to treatment of either single miRNA with cisplatin [113].

A search of the Australian New Zealand Clinical Trials Registry (ANZCTR) and National Institutes of Health Clinical Trials database (clinicaltrials.gov) did not show any active or completed clinical trials involving the use of miRNA mimics or anti-miRs in HGSOC chemotherapy regimens.

## 5. Conclusions

The complexity and constantly evolving nature of the epigenetic features of HGSOC have been a major challenge to developing effective epigenetic therapeutics with tolerable toxicity profiles. The changes in DNA methylation, histone deacetylation, and miRNA expression that occur as HGSOC develops resistance to standard-of-care chemotherapies have been difficult to determine and target for effective therapeutic development. The most promising area of therapeutic potential for treatment-resistant HGSOC is DNMTi in combination with traditional chemotherapy. The results of the early phase 2 clinical trials have provided evidence that the combination should be followed up in larger studies. As the field of HDACi develops further, there is also promise that the combination of HDACi and chemotherapy will provide an alternate treatment option once resistance to standard-of-care chemotherapy occurs.
